# The Macrophage Iron Signature in Health and Disease

**DOI:** 10.3390/ijms22168457

**Published:** 2021-08-06

**Authors:** Christina Mertens, Oriana Marques, Natalie K. Horvat, Manuela Simonetti, Martina U. Muckenthaler, Michaela Jung

**Affiliations:** 1 Department of Pediatric Hematology, Oncology and Immunology, University of Heidelberg, INF 350, 69120 Heidelberg, Germany; oriana.marques@med.uni-heidelberg.de (O.M.); natalie.horvat@embl.de (N.K.H.); Martina.Muckenthaler@med.uni-heidelberg.de (M.U.M.); 2 Molecular Medicine Partnership Unit, 69120 Heidelberg, Germany; 3 European Molecular Biology Laboratory (EMBL), Collaboration for Joint PhD Degree between EMBL and the Faculty of Biosciences, University of Heidelberg, 69117 Heidelberg, Germany; 4 Institute of Pharmacology, Medical Faculty Heidelberg, Heidelberg University, INF 366, 69120 Heidelberg, Germany; manuela.simonetti@pharma.uni-heidelberg.de; 5 Institute of Biochemistry I, Faculty of Medicine, Goethe-University Frankfurt, 60590 Frankfurt, Germany

**Keywords:** macrophage polarization, iron metabolism, disordered iron metabolism

## Abstract

Throughout life, macrophages are located in every tissue of the body, where their main roles are to phagocytose cellular debris and recycle aging red blood cells. In the tissue niche, they promote homeostasis through trophic, regulatory, and repair functions by responding to internal and external stimuli. This in turn polarizes macrophages into a broad spectrum of functional activation states, also reflected in their iron-regulated gene profile. The fast adaptation to the environment in which they are located helps to maintain tissue homeostasis under physiological conditions.

## 1. Iron and Macrophage Polarization—A General View

Macrophages (MΦ) are innate immune cells located in every tissue of the body. They are involved in processes as diverse as inflammation, development, tissue remodeling, and metabolism. MΦ show remarkable plasticity depending on signals derived from the organ niche in which they are located [1]. Through their capacity to engulf and digest foreign particles, such as pathogens, tissue debris, or damaged cells, they play a critical role in maintaining tissue homeostasis. In many organs, tissue-resident MΦ are derived from precursor cells of fetal origin that are self-renewing and long-lived, and maintain a homeostatic pool without contribution of infiltrating monocytes [2]. In some tissues, however, monocyte-derived cells with a shorter lifespan can replace tissue-resident MΦ.

MΦ show enormous plasticity and functional diversity, which allow for rapid adaptation of the MΦ phenotype to varying stimuli within an inflammatory environment [3]. Two extreme phenotypes, namely the classical and alternative MΦ phenotypes, were identified within a broad continuum of different possible MΦ activation states. Classically activated MΦ show a potent pro-inflammatory profile and play a critical role in host defense against microbes, as well as tumors [4]. This MΦ subpopulation activates the production and secretion of pro-inflammatory mediators such as tumor necrosis factor (TNF)-α; interleukin (IL)-1β, IL-6, IL-12, and IL-23; and reactive oxygen (ROS) and nitrogen (Nos) species [5,6], and is capable of presenting antigens to T cells. In contrast, alternatively activated MΦ represent a functionally opposite phenotype, with enhanced secretion of anti-inflammatory cytokines and chemokines, expression of specific phagocytic receptors, production of extracellular matrix, and growth factors that are pivotal for tissue remodeling [7]. They are key to the resolution of inflammation and to combat extracellular parasites.

Taking this functional diversity into account, it is not surprising that their polarization profile may also be reflected by diverse iron-associated phenotypes [8]. The steps of iron recycling from erythrocytes in MΦ comprise uptake, storage, and release of iron. In line with a healing and supportive role, alternative MΦ express high levels of transferrin receptor (TfR)1 [9] and low levels of ferritin, and contribute to tissue homeostasis by recycling red blood cells or iron in the form of heme, hemoglobin, and hemopexin, as well as by clearing away dead cells [10,11]. High levels of ferroportin (FPN1) coupled with clearance functions suggest that alternative MΦ retain low levels of iron, but actively provide iron to tissue cells, thereby acting as nutritive suppliers [12,13]. On the contrary, iron-loaded MΦ have low levels of FPN1, as well as high levels of TFR1 and ferritin. Iron-retaining MΦ also show enhanced expression of pro-inflammatory cytokines, such as IL-6, IL-1β, and TNFα, which in turn are essential for the sequestration of iron in situations where iron maybe detrimental; i.e., during infection [10]. The special way that MΦ handle iron is also a significant factor in reinforcing their activation status, thereby acting as a phenotypic driver [10].

By applying iron to MΦ, unstimulated bone-marrow-derived MΦ (BMDMs) can be activated to a classical-like phenotype [14,15]. The importance of iron in promoting this phenotype has also been demonstrated for classical polarization of MΦ, where classical activation was attenuated in MΦ lacking an iron source [16]. Moreover, studies have shown that applying NTBI to alternative-like MΦ can initiate a phenotypic switch towards an classical-like phenotype [15,17]. While these results obtained in cultured cells may show more pronounced effects compared to in vivo situations, these experiments show the potential of iron in dictating MΦ function. The degree of activation critically depends on the iron source.

In this review, we will focus on the important role of MΦ in iron metabolism, covering iron uptake, acquisition, storage, and release. As a consequence of phagocytosing damaged erythrocytes and other cell types that contain iron, they serve as an iron store able to supply iron for erythropoiesis, as well as to neighboring cell types in need of iron to stimulate their proliferation and growth; i.e., during recovery phases after severe tissue damage. As iron is a critical component of enzymes of the electron transport chain that assures cellular energy production and proteins involved in DNA synthesis, or for metabolic enzymes, iron is essential in all cell types.

## 2. MΦ in Systemic Iron Homeostasis

More than half of the iron contained in the adult human body (approx. 4 g) is found in hemoglobin within erythrocytes [8]. The recycling of iron from aging or damaged red blood cells in MΦ contributes most of the iron required for erythropoiesis. Only 1–2 mg of iron/day is absorbed from the diet by the gastrointestinal (GI) system, compensating for iron losses due to desquamation and bleeding, whereby approximately 25 mg of iron/day is supplied by MΦ as a consequence of erythrophagocytosis (reviewed by Muckenthaler et al. in [18]).

Erythrocytes that reach their maximum life span of approximately 120 days are recycled by highly specialized splenic red pulp MΦ [19,20]. These MΦ are produced by progenitors that migrate to the spleen perinatally, involving transcriptional programs induced by the transcription factors SPI-C and PPARγ [21,22]. Their primary functions include the filtering of microorganisms and senescent red blood cells.

Aging-related changes in erythrocytes, including alterations and membrane clustering of the highly abundant membrane protein Band 3, the appearance of phosphatidyl serine (PS) on their outer leaflet of the outer membrane, or increased membrane rigidity, are recognized by specific receptors expressed by MΦ [23,24]. In addition, infections, hemoglobinopathies, or alterations in metabolism may induce a cell-death program in red blood cells called eryptosis, a state recognized by MΦ [20]. The ingested erythrocyte enters the phagocytic vacuole, where exposure to ROS or hydrolytic enzymes causes release of hemoglobin and heme into the vacuolar fluid. While some studies indicate that iron is released from heme in the phagolysosome and transported to the cytoplasm as iron, more recent evidence suggests that heme is transported across the phagolysosomal membrane by the heme transporter heme-responsive gene-1 (HRG-1) [25]. Heme-oxygenase (HO-1), a membrane-bound enzyme, is essential for heme degradation of iron, biliverdin, and carbon monoxide [26]. A lack of HO-1 in mouse models causes iron accumulation in spleen and liver of mice and low serum iron availability in the plasma—consistent with a defect in iron recycling [26,27].

Conditions such as hemoglobinopathies, excessive exercise, or infections may trigger uncontrolled lysis of erythrocytes in the vasculature. As a consequence, hemoglobin or heme is released into the bloodstream [28]. Hemoglobin is recognized by haptoglobin, and the resulting complex is endocytosed by hepatocytes and MΦ via the cluster of differentiation (CD)163 receptor [28]. Oxidation of hemoglobin converts heme-bound ferrous iron to ferric iron (hemin), which will be recognized by hemopexin. Hemopexin–heme complexes bind the CD91 receptor and are internalized into MΦ and hepatocytes, where iron is released via the activity of HO-1 [29].

Iron released from heme into the cytoplasm initially enters the redox-active labile iron pool (LIP). Heme and/or iron activate the nuclear factor erythroid 2-like (NRF2), a transcription factor that assures coordinated iron recycling via stimulating transcription of the iron storage protein ferritin and iron export via the sole known iron exporter FPN1 [30,31]. In addition, iron levels activate ferritin and FPN1 translation by inhibiting its repression by the iron responsive element (IRE)/iron responsive protein (IRP) regulatory system [32].

FPN1 is a member of the major facilitator superfamily of transporters, and acts together with a ferroxidase, ceruloplasmin (CP), that converts the exported ferrous to ferric iron for binding to the plasma carrier protein transferrin (Tf) [33]. Tf-bound iron can be taken up by cells that express TfR1 to satisfy their iron demand. Importantly, the process described here may not only occur in MΦ specialized for iron recycling, but also in MΦ infiltrating hemorrhagic tissues (e.g., during cancer or a hemorrhagic stroke). Iron export from MΦ via FPN1 is a highly regulated process involving transcriptional, post-transcriptional, and post-translational mechanisms. The iron-regulated hormone hepcidin plays a critical role in the control of FPN1 cell surface expression. Hepcidin is produced by the liver in response to high iron levels and inflammation. Under these conditions, hepcidin binding to FPN1 triggers FPN1 ubiquitinylation and degradation, and thus prevents iron export from MΦ [34]. The same mechanism controls dietary iron uptake in the duodenum. Increased hepcidin levels in the liver due to high iron availability thus act in a negative feedback manner to prevent additional iron uptake. Under inflammatory conditions, high hepcidin levels trigger iron retention in MΦ, whereby iron availability in the plasma is reduced [35]. This is considered an innate immune mechanism that restricts iron; e.g., for growing microorganisms. However, if the infection persists, too little iron will be available for red blood cell synthesis, and as a result, anemia of inflammation will develop. We recently demonstrated that MΦ also decrease FPN1 transcription in response to inflammation to retain iron. Patterns recognized by the toll-like receptors (TLR) 2 and 6 convey signals to inhibit *Fpn* mRNA expression independently of hepcidin [36]. In contrast, TLR 4-mediated signals decrease *Fpn* mRNA levels and induce hepcidin at the same time [37]. On the other end of the spectrum, high iron demand for erythropoiesis and hypoxic conditions reduce hepcidin levels in the liver, allowing for efficient iron export out of MΦ to supply iron to the bone marrow to stimulate erythropoiesis.

MΦ not only phagocytose aging red blood cells, but also engage in the elimination of apoptotic cells in a process called programmed cell removal or efferocytosis [38]. A combination of “find-me” and “eat-me” signals exposed by apoptotic cells triggers their phagocytosis. Once a physical connection is established between apoptotic cells and MΦ, signalling events are triggered, causing internalization of the apoptotic particle in the phagolysosome. During this process MΦ produce mainly anti-inflammatory cytokines such as transforming growth factor (TGF)-b, prostaglandin E2 (PGE2), or interleukin (IL)-10, and suppress the production of proinflammatory cytokines such as tumor necrosis factor (TNF)-a, IL-1, IL-12, and IL-8. This process avoids tissue inflammation. Any disturbance of efferocytosis can cause disorders such as inflammatory and autoimmune diseases, atherosclerosis, and cancer. How MΦ handle iron during the efferocytosis process requires further investigation [39].

Taken together, MΦ play a central role in balancing iron levels in the plasma to supply sufficient amounts of iron to all cell types, and at the same time, prevent iron excess that cause cellular toxicity and ferroptosis mediated by Fenton chemistry.

## 3. Gastrointestinal System

### 3.1. Gut MΦ

Former belief advocated for tissue MΦ being generated from blood monocytes derived from bone marrow progenitors. However, recent developments demonstrated that many tissue MΦ exist independently from conventional hematopoiesis, and rather arise from yolk sac or fetal liver precursors. In contrast, it appears that the intestine is initially seeded by embryo-derived MΦ that are substituted, over time, by MΦ generated through hematopoiesis. In the steady state, the largest population of resident MΦ in the body is found in the intestine. Like their counterparts in other tissues, GI MΦ are highly phagocytic and participate in tissue remodeling and removal of cellular debris. Gut MΦ are considered key players in the maintenance of gut homeostasis, as they produce crucial cytokines and factors involved in the proliferation and differentiation of epithelial progenitors and enteric neurons to prevent excessive inflammation. This is of crucial importance, as the intestine has the highest bacterial burden in the body. Exposure of these MΦ to foreign antigens does not result in overt inflammatory responses, likely due to their priming to commensal microbiota (reviewed in [40]). An imbalance in MΦ activation skews this delicate equilibrium in favor of loss of tolerance towards common gut antigens, resulting in chronic inflammation observed in patients with inflammatory bowel disease (IBD) [41]. Most knowledge about the iron metabolism of the gut microenvironment by MΦ was gained by studying pathological inflammation, for which anemia is a consistent clinical feature. Anemia is caused either by impaired iron uptake, as a result of gut inflammation, or by blood loss in the GI tract that is not compensated by duodenal iron absorption (see section above) [42,43,44]. Moreover, local and systemic inflammation may contribute to the establishment of functional iron deficiency, further adding up to form the above-mentioned clinical picture. In this setting, GI MΦ are of particular importance, since they are major local producers of cytokines, thus creating the inflammatory microenvironment [45,46,47], and they are also the main iron-retaining cells due to downregulation of the iron exporter FPN1 under inflammation [36,48]. Moreover, impairment of the immune response has been reported both during iron deficiency and overload (reviewed in [49,50]), likely reflecting a narrow range for cellular iron levels promoting proper immune responses, which may have consequences for MΦ immunosurveillance. In fact, several studies have described a link between *Helicobacter pylori* stomach infection and iron-deficiency anemia [51,52,53], but a clear physiological mechanism and potential relationship with MΦ iron handling remains unexplained. Likewise, physiological mechanisms regulating iron absorption in the duodenum and proximal jejunum are directed towards enterocytes, but whether interactions with other cell types in the GI tract influence the process has not been studied so far. Interestingly, in a recent work, Bessman and coworkers identified type 2 conventional DCs (cDCS2), and not MΦ, as the main myeloid producers of hepcidin in the colon in IBD. In this setting, hepcidin is required for intestinal repair, as demonstrated by abnormal colonic architecture and altered composition of the intestinal microbiota in *Hamp*^∆CD11C^ mice treated with dextran sodium sulfate (DSS) in comparison with treatment-matched controls. Nonetheless, they also demonstrate that FPN1-expressing MΦ are main targets for hepcidin-mediated mucosal healing [54]. As dendritic cell-derived hepcidin only appears to be induced in the inflamed intestine, the potential role for the local regulation of iron distribution by hepcidin derived from myeloid cells in the gut still remains unidentified.

### 3.2. Other MΦ Populations in the Gastrointestinal System

Hepatic-resident MΦ (Kupffer cells [KCs]), in contrast to gut MΦ, are derived from fetal liver precursors, and constitute the most abundant type of immune cells in the liver. Besides the embryo-derived KCs, the liver is also populated by the liver capsular MΦ, arising from adult circulating monocytes [55]. KCs perform primary scavenging, phagocytic, and immune-surveillance functions in the liver, and are able to modulate the liver’s regulatory functions in terms of iron homeostasis. This is best represented by the ability of KCs to downregulate hepcidin levels, as in vivo liposome-encapsulated clodronate depletion of KCs results in increased hepcidin expression, with a concomitant reduction in serum iron levels [56]. KCs are located within the lumen of liver sinusoids, and this privileged location allows for contact with, among others, nutrients, pathogen-associated molecules, and toxins transported from the GI tract via the portal vein.

Although KCs are highly tolerogenic, some of these products will still lead to their activation [57]. This is particularly important for the detection of pathogenic ligands, given that KCs are the first immune cells in the liver to come in contact with this type of product, and also are the main liver producers of the hepcidin-activating cytokine IL-6 [48,57]. Despite that, other studies have reported that KCs are not required for the induction of hepcidin upon iron overload or inflammation [58,59]. Another major role of KC in the liver is erythrophagocytosis. After the spleen, the liver is the second most important organ for red blood cell (RBC) and iron recycling [60], with recent studies reporting that, under pathological conditions causing damage of RBCs in the bloodstream, the liver is the primary site for RBC clearance [61,62], offering another layer of protection against an acute heme insult. Several studies have also established a role for KCs in the development of liver diseases, such as alcoholic liver disease (ALD), non-alcoholic steatohepatitis (NASH), fibrosis, or hepatocellular carcinoma (HCC) [63]. Reported associations between the functional phenotype of KCs and the progression of hepatic diseases reflect a wider spectrum of MΦ polarization than reported for most diseases. Whether disease progression and/or severity is influenced by the iron status of KCs, as has been demonstrated for KC iron loading in experimental ALD [64], remains a topic of interest.

Furthermore, tissue-resident MΦ also populate the exocrine and endocrine pancreas, where they perform functions related to immune surveillance and likely angiogenesis or lymphogenesis, depending on their location [65]. Origin and phenotype of pancreatic MΦ differs according to their microenvironment. MΦ in the islets of Langerhans have been reported to be derived from conventional definitive hematopoiesis and display an inflammatory phenotype, while MΦ in the exocrine pancreas may be derived either from primitive or definitive hematopoiesis and are mostly tolerogenic [66]. The pro-inflammatory phenotype of MΦ in the islets of Langerhans, crucial for the constant probing of the microenvironment in their vascular beds, has been deemed particularly detrimental in pathological conditions such as diabetes [65,67,68]. In the context of tissue iron regulation, β cells on the islets are particularly susceptible to lipotoxicity under iron deficiency [67], and it has been postulated that MΦ could supply β cells with the necessary iron to protect them from this type of assault [68]. However, in mouse models of iron overload, iron has been shown to be stored mainly in the exocrine pancreas, more specifically in acinar cells [69,70,71]. Although it may be tempting to hypothesize that the different intraregional MΦ phenotypes (and iron handling) may contribute to the observed differences in iron loading in the pancreas, experimental validation still awaits.

### 3.3. MΦ and Malignancies of the Gastrointestinal System

As iron is an essential nutrient for (malignant) cell proliferation, iron intake and systemic iron levels have been historically considered risk factors for colorectal cancer (CRC) [72,73,74]. As reported for other cancer cell types, CRC cells display an “iron-retaining” phenotype with increased expression of iron importers and decreased expression of iron exporters in advanced tumors [75,76], correlating with elevated iron content in CRC in comparison with normal adjacent tissues [75,77]. Accordingly, mice on a low-iron diet developed fewer colon tumors in comparison with mice on an iron-replete diet [77,78]. As chronic inflammation is a major hallmark of CRC, it is not surprising that the presented risk for CRC in IBD patients is substantially higher [79,80]. Iron is thought to promote tumorigenesis in an inflammatory setting, as dietary iron supplementation worsens chronic inflammation and promotes tumor development in CRC mouse models [81,82]. In the tumor microenvironment, MΦ represent the largest infiltrating leukocyte population, influencing the formation, growth, and metastasis of tumors through their interaction with cancer cells. Despite this general “dogma”, the contribution of MΦ to the development of CRC is less clear, and has been attributed to differences in MΦ characterization and location within the tumor [83]. In general terms, the presence of pro-tumorigenic MΦ in the tumor microenvironment has been considered an adverse prognostic marker of survival for several cancer types [84,85]. These MΦ are characterized by the surface expression of CD163, a hemoglobin scavenger receptor and therefore specialized in the uptake of heme-bound iron [86,87]. This parallels the current concept that these MΦ may further contribute to tumor growth and development due to their iron-recycling capacity, promoting iron release towards cancer cells via high expression of the iron exporter FPN1 and carrier lipocalin-2 (LCN-2) [15,88,89]. Things are further complicated in the case of CRC, with several studies demonstrating that increasing numbers of MΦ infiltrating the tissue microenvironment correlated with improved survival in patients [90,91], which may reflect higher numbers of iron-loaded, pro-inflammatory, and anti-tumorigenic MΦ. Recent studies have shed light on this controversy by demonstrating that MΦ infiltration may exert different effects on tumor growth and progression, depending on the tumor site and hypoxia conditions (reviewed in [88]), but to the best of our knowledge, to this date, the “iron phenotype” of these MΦ has not been identified in CRC, nor correlated with clinicopathological markers of behavior and progression.

## 4. Cardiovascular System

### 4.1. Cardiac MΦ Populations

Different MΦ subpopulations have been described in the heart involving tissue-resident MΦ, embryonically-derived MΦ, and infiltrating monocyte-derived MΦ [89,92,93]. Cardiac-resident MΦ originate from self-renewing embryo-derived populations, and can be classified in various subsets. MΦ precursors have been shown to seed the embryonic heart beneath the epicardium [94]. Their property to self-renew faints with aging, and monocyte-derived MΦ gradually substitute the embryo-derived subpopulation [93]. The diversity of the cardiac MΦ phenotype is constantly shaped to ensure tissue homeostasis by fulfilling tissue-specific functions, ranging from homeostatic functions, such as clearance of cellular debris, up to major roles in tissue immune surveillance and resolution of inflammation. Recently, it was also shown that cardiac MΦ modulate the electrical activity of cardiomyocytes and are able to facilitate electrical conduction through the distal atrioventricular node [95]. These diverse properties are attributed to distinct MΦ subtypes that are reflected by their polarization state.

All populations that have been identified in the mouse heart express varying levels of lymphocyte antigen 6 (Ly6C) and major histocompatibility complex (MHCII) [96,97]. In the healthy heart, yolk-sac-derived resident MΦ negative for Ly6C and C-C chemokine receptor type 2 (CCR2) predominate together with embryonic progenitors that are not replenished by circulating monocytes under steady-state conditions [89,98]. This subgroup of MΦ contains MHCII-low and -high subsets. CCR2+ MΦ are replenished by blood monocyte recruitment and local proliferation, whereas CCR2– MΦ are repopulated by local proliferation [23]. Resident MΦ in the healthy state are anti-inflammatory and express a gene profile similar to alternative MΦ, promoting angiogenesis and tissue repair to maintain cellular homeostasis. Under physiological conditions, resident cardiac MΦ remove senescent and dying cells in the myocardium. Considering their phagocytic activity, cardiac-resident MΦ are polarized towards an anti-inflammatory state, and it may be speculated that this subpopulation releases iron to the cardiac environment to maintain cellular homeostasis.

In contrast, infiltrating pro-inflammatory monocyte-derived MΦ (Ly6C+ CCR2− and Ly6C+ CCR2+) promote tissue injury and death by substitution of the resident MΦ subpopulation [89,98,99,100]. After injury under inflammatory conditions, there is evidence that resident MΦ are substituted by splenic or bone-marrow-derived MΦ (BMDMs) [101,102]. Due to their polarization profile, monocyte-derived infiltrating MΦ may sequester iron and thereby foster cardiac injury and scar formation. Experimental ablation of resident MΦ has shown that BMDMs are able to replace the resident MΦ population under some conditions [103,104]. Depletion of resident cardiac MΦ in a murine model of myocardial infarction resulted in an increased infarct area, reduced left ventricular (LV) systolic function, and aggravated LV remodeling [99]. So far, there is not much known about the cardiac MΦ iron phenotype in health and disease. However, it is well known that cardiac iron levels must be tightly regulated; cardiomyocytes are highly susceptible to iron-induced cell death, known as ferroptosis. However, they also require high amounts of iron for energy production in mitochondria by iron-containing enzymes. This makes iron an important player and an additional risk factor for cardiovascular disease. Metabolic disturbances can lead to changes in the myocardial structure and cardiac function by inducing a smoldering inflammation and, in turn, oxidative stress, mitochondrial dysfunction, endoplasmic reticulum stress, and impaired calcium handling [105].

### 4.2. Role of Iron and MΦ in Cardiac Inflammation and Disease

When the heart is under stress or injured, it undergoes cardiac remodeling, involving structural and functional changes [106]. These include cardiac hypertrophy [107], fibrosis [108], apoptosis [109], and an altered metabolism. In the first 24 h after coronary ligation in mice, half of all monocytes recruited to the heart derive from splenic reservoirs [110]. After entering the cardiac tissue, monocytes differentiate to MΦ that are recruited by the CCL2/CCR2 axis [111,112] to produce both pro-inflammatory and anti-inflammatory mediators, phagocytose dead cells, and promote angiogenesis and scar formation. Directly after an acute ischemia/reperfusion injury, the inflammatory response of MΦ is required for clearance of the necrotic myocardium by phagocytosis [113]. The recruitment of reparative monocytes (Ly6C−) helps to resolve inflammation and promote wound healing [114]. The highest level of this inflammatory MΦ subtype can be found approximately 3 days after injury [98]. At day 5–7 [115,116], MΦ populations reach their maximum in the infarct zone predominantly with a pro-inflammatory phenotype. After cardiac injury, the microenvironment becomes hemolytic due to ROS causing cellular debris due to disruption of red blood cells, as well as the breakdown of collagen. It may be speculated that in this microenvironment, MΦ may become iron-loaded by clearing the cardiac tissue. Additionally, iron itself could trigger inflammation, as ferric ammonium citrate was shown to induce MΦ-dependent IL-1β secretion and trigger ventricular arrhythmias in mice [117]. IL-1β is a regulator of the inflammatory response occurring after myocardial infarction, and is involved in the recruitment of immune cells, cytokine production, and extracellular matrix degradation. The underlying inflammatory signaling cascades of these cytokines facilitate an early response to myocardial injury, and entails mitochondrial ROS overproduction [118]. ROS-mediated mitochondrial dysfunction and lysosomal membrane permeabilization trigger inflammasome activation via hypoxia inducible factor (HIF) [105]. HIF increases TfR1 expression at the transcriptional level, leading to an increased iron accumulation and enhanced oxidative damage by ROS [119]. Iron overload occurring during hereditary hemochromatosis or cardiac hemorrhage increases the LIP and contributes to iron-mediated cell death of cardiomyocytes [120] and cardiac dysfunction [121]. In turn, cardiomyocyte death and cardiac dysfunction cause an increased accumulation of lipid peroxides [121]. For example, it was shown that high serum iron levels are correlated with severity of coronary artery disease [122]. During the course of atherosclerosis, iron is deposited in lesions in the form of hemoglobin/hemin occurring during hemolysis in the inflammatory microenvironment or intraplaque hemorrhage, which further contributes to intracellular accumulation of iron and inhibition of phagocytosis [123]. In humans, it has been shown that atherosclerotic lesions contain high amounts of ferritin light and ferritin heavy chain, highlighting the accumulation of iron [124]. The LIP of circulating monocytes was positively correlated with the TfR1/ferritin ratio and hepcidin levels, as well as the progression of atherosclerosis and arterial stiffness [125]. It may be speculated that the LIP of monocytes could be an indicator of atherosclerotic conditions in arteries [120]. Additionally, heme, as well as iron, were found to contribute to LDL formation in different cell types, such as endothelial cells [123], smooth muscle cells [126], and MΦ [127]. Nevertheless, systemic parameters measured in the blood of patients such as ferritin and Tf also could be useful markers for elevated serum iron concentrations associated with increased syntax score and atherosclerosis severity [122]. A relation between plasma iron values and the intracellular iron content is not fully understood. Inflammatory mediators contribute to atherosclerosis, and additionally increase the expression of hepcidin. In turn, serum iron levels are reduced by decreased duodenal iron absorption and iron sequestration in MΦ. In contrast, hepcidin deficiency is protective for atherosclerosis by reducing MΦ iron and the inflammatory phenotype [128].

Iron deficiency during heart failure is highly prevalent, affecting up to 50% of patients [129,130]. Iron deficiency results in left ventricular hypertrophy and dilatation, and cardiac fibrosis. At the molecular level, if iron uptake is impaired by cardiomyocyte-specific knockout of TfR1 in mice, severe heart failure is observed due to a failure of mitochondrial respiration [131]. In early clinical studies, iron deficiency in heart failure patients was only considered clinically relevant in combination with anemia. More recent studies demonstrated that reduced hemoglobin levels were the result of a process starting with the gradual depletion of iron stores [132]. Even in the absence of anemia, iron deficiency is common in heart failure patients [133,134] and is an independent predictor of poor outcome [134]. The pathophysiology of why iron deficiency correlates with poor prognosis in heart failure patients and how iron supplementation affects these patients, especially at the cellular level, is incompletely understood. During heart failure development, inflammation plays a central role, whereby the inflammatory response enables regenerative processes. In the early stages, Ly6C+ monocytes infiltrate the heart and differentiate into inflammatory MΦ, promoting adverse left ventricular remodeling [135]. Considering the abundance of this MΦ subpopulation and the polarization profile, it may be speculated that iron is trapped inside immune cells, causing cellular iron deficiency, and thereby contributes to cardiac injury. Interestingly, the systemic iron status does not necessarily correlate with the cellular iron status [136]. A reduced cardiac iron content may occur despite normal systemic iron stores [137]. In turn, cardiac mitochondria may be iron-overloaded in heart failure patients despite systemic iron deficiency [138].

## 5. Kidney

### 5.1. MΦ Phenotypes in Acute and Chronic Renal Pathologies

MΦ are involved in promoting kidney injury, but also in fostering resolution of inflammatory disease, as well as renal repair [139,140]. MΦ constitute one of the major infiltrating immune cell populations following renal damage, whereby their function largely depends on their phenotypic characteristics and their activation status [141]. We and others found that MΦ adopt an inflammatory phenotype with enhanced expression of iNOS [142] and pro-inflammatory cytokines, including IL-1β and TNF-α [143], during early phases after acute renal injury, whereas during later phases of resolution and recovery, predominately anti-inflammatory, arginase-1 (Arg1)-expressing, mannose-receptor-positive MΦ were identified. These observations were impressively shown by MΦ depletion studies, in which inhibition of MΦ infiltration not only blocked injury development during acute phases of injury, but also inhibited renal repair mechanisms in subsequent recovery and resolution phases [144]. Whereas MΦ depletion before the onset of acute injury protected against the loss of renal function and tubular injury upon acute kidney injury (AKI), the infusion of pro-inflammatory MΦ was able to restore the AKI injury profile [145,146]. Accordingly, we found that ex vivo genetically modified anti-inflammatory MΦ clearly protected against ischemia-dependent functional decline and kidney inflammation [143,147].

Therefore, it may be speculated that the control of the local MΦ phenotype plays a decisive role in disease outcome. Ly6C+ monocytes are attracted to the inflamed kidney after acute injury, and migrate to the site of damage via CCR2 and CX3C chemokine receptor 1 (CX3CR1) [145,148]. It is likely that high-mobility group box 1 (HMGB1) is released after ischemic renal injury, which further promotes active MΦ recruitment [149]. While MΦ accumulate during the acute phase of renal injury, their local proliferation is considered a critical hallmark of chronic kidney disease (CKD) [150,151]. Monocytes, which are derived from the bone marrow, are the precursors of differentiated MΦ populations within the kidney. Therefore, interfering with colony stimulating factor 1 receptor (CSFR1) was shown to significantly block MΦ accumulation and proliferation within the kidney due to its inhibitory function regarding monocyte maturation and proliferation in the bone marrow [152,153]. Moreover, CCL2, which binds to CCR2, is involved in monocyte migration towards the inflamed sites of renal injury, whereby the blockade of CCL2 significantly attenuated both glomerular and interstitial MΦ infiltration and accumulation [154,155,156].

Additionally, CX3C chemokine ligand (CX3CL)1 and CX3CL16, as well as MΦ inhibitory factor (MIF), are implicated in MΦ recruitment during the development of renal pathologies [157,158]. Upon differentiation of monocytes, MΦ are activated and polarized by the predominant inflammatory status of the tissue during acute injury. Pro-inflammatory cytokines and DAMPs foster the pro-inflammatory, tissue-destructive MΦ phenotype [159,160,161]. Moreover, the crosstalk of MΦ and renal cells plays a pivotal role in the maintenance of the MΦ polarization status. This includes the production and secretion of cytokines from both MΦ and renal cells, but also exosomal delivery of RNA and miRNA, which massively impacts MΦ polarization, as well as the extent of inflammatory outcome and disease progression [162,163]. In this sense, it was also shown that, even if absolute numbers of infiltrated CD64+ MΦ are similar, levels of cytokine activation within the kidney are decisive for MΦ polarization and, accordingly, for their subsequent impact on renal disease development or repair. Renal parenchyma-derived DAMPs such as DNA, HMGB1, or C reactive protein (Crp) further enhance the accumulation of pro-inflammatory MΦ, which, in turn, exacerbate renal injury [164,165,166,167]. Therefore, even if pro-inflammatory MΦ are needed during early phases of injury to remove inflammatory dead cells, such as neutrophils, and to clear secreted DAMPs, a prolonged or uncontrolled activation of pro-inflammatory MΦ not only fosters massive tissue injury and inflammation, but also delays renal repair mechanisms.

During later phases of disease development, a conversion of pro-inflammatory towards anti-inflammatory MΦ population takes place [168]. Anti-inflammatory MΦ, which are characterized by high expression of Arg1, dectin-1, and mannose receptor (CD206), play a pivotal role for the regeneration of damaged epithelial cells and proliferative recovery of the tissue architecture [142,169]. Moreover, they are highly phagocytic and massively clear intraluminal debris and apoptotic cells within the tissue. Additional beneficial effects rely on their ability to activate regulatory T cells, as well as the control of the inflammatory response [170,171,172]. However, again, tightly controlled MΦ activation is pivotal to avoid extensive repair with subsequent fibrosis development [173]. Recently, it was shown that the activation of mineralocorticoid receptors (MR) is involved in the control of the anti-inflammatory MΦ phenotype, whereby the transition of acute injury towards chronic injury might be controlled [174]. Additionally, deposited immunoglobin may add to the recruitment and activation of MΦ, which is mainly accomplished through the fragment receptor (FcR) [175]. Along these lines, another source of MΦ that may be implicated in the outcome of tissue inflammation and renal disease development, are resident renal MΦ. Despite very limited information about this MΦ population, it was recently shown that immunoglobulin gamma Fc region receptor (Fcgr)4− and Fcgr1+ resident MΦ are implicated in renal repair through the activation of the Wnt pathway [176,177]. Interestingly, these resident MΦ do not adopt a clear pro-inflammatory or anti-inflammatory phenotype, but rather show a characteristic and uniquely mixed phenotypic signature.

However, taking into account the positive effects of anti-inflammatory MΦ during renal recovery from acute injury, their uncontrolled activation and function may lead to renal fibrosis. Interestingly, a positive correlation was found between anti-inflammatory CD163+ MΦ and the severity of kidney fibrosis in immunoglobulin A (IgA) nephropathy, type 2 diabetes, and chronic kidney allograft injury [178,179,180]. Along these lines, experimental murine models of CKD showed that anti-inflammatory MΦ are critically involved in disease progression during glomerulonephritis, and interstitial fibrosis during nephrotoxic nephritis [181,182].

### 5.2. Renal Iron Handling—MΦ versus Tubular Epithelial Cells

Interestingly, it has been widely recognized that iron handling by both renal MΦ and the epithelium may be a pivotal factor in determining the fine balance between tissue damage versus recovery [183,184]. It was suggested that these two compartments act in an orchestrated manner in order to coordinate the renal response towards injury and foster the subsequent recovery. These observations are not surprising due to the critical role of the kidney in reabsorbing iron that is bound to specific proteins, including Tf, ferritin, or LCN-2. This is accomplished by the expression of non-specific megalin and cubilin receptors at the apical plasma membrane of proximal tubular epithelial cells [185,186,187,188], from which iron is recycled into the circulation in order to prevent the loss of filtered iron by urinary excretion [144,189]. Despite the very prominent role of the kidney for iron homeostasis, there is still very limited information about mechanistic details of renal iron recycling and transport. Moreover, it is still not clear how different renal compartments interact with regard to differences in the iron phenotype. It has been demonstrated, for more than 20 years now, that renal ischemia/reperfusion injury promoted the formation of catalytic iron, whereby cellular damage was induced through the activation of oxidative stress pathways [190]. Recently, this type of cell death linked to the action of oxidized iron was identified as ferroptosis, taking place upon inhibition of the membrane glutamate/cysteine exchange, as well as the depletion of cellular antioxidants [191]. Along these lines, iron-mobilizing molecules such as LCN-2, ferritin, hemopexin, and haptoglobin were highly induced upon renal injury [192], all showing a renoprotective function upon infusion.

It is widely accepted that the application of LCN-2 diminishes renal injury and enables renal regeneration [147,151,175,193,194]. Recently, we found that LCN-2 fulfills different biological functions according to its iron-loading status and its cellular source during sepsis-induced kidney failure [195]. Interestingly, LCN-2 was produced and secreted from renal tubular epithelial cells in its iron-free form, which was associated with renal damage. In contrast, increased levels of MΦ-derived LCN-2 appeared in the iron-loaded form and significantly correlated with renal recovery markers. With regard to LCN-2, we previously showed that anti-inflammatory, LCN-2-overexpressing MΦ that were infused during the early onset of acute ischemic injury not only protected from renal tissue destruction and the decline of renal function, but also promoted renal regeneration [143]. Additionally, a recent study from our group determined that tubular epithelial cells took up MΦ-secreted, iron-loaded LCN-2 in an in vitro cisplatin-induced injury model, which correlated with cellular proliferation and recovery [196]. Moreover, our own observations are in line with other studies, suggesting that the renoprotective effects of LCN-2 may be due to its ability to serve as an iron transporter [197]. Taking into account the action of ferritin heavy chain as a ferroxidase enzyme, promoting the storage of inert-state iron, ferritin heavy is critical for the protection of proximal tubular cells against cisplatin-induced, as well as obstructive, kidney injury [193,198]. Ferritin heavy chain deficiency in proximal tubules is associated with enhanced tubular damage and obstructive disease, as well as increased MΦ infiltration and pro-inflammatory activation. On the contrary, myeloid ferritin heavy chain deficiency led to increased levels of HO-1 expressing MΦ, associated with reduced levels of fibrosis in an unilateral ureteral obstruction (UUO) kidney-injury model [193]. This is interesting in the sense of the observation that iron-regulated genes may adopt different roles and functions with regard to acute versus chronic disease progression. Specifically, HO-1 expression adopts a critical role in MΦ iron handling due to its heme-degrading function upon erythrophagocytosis, with a loss of myeloid HO-1 being associated with dysregulated heme recycling, iron-induced oxidative stress and, consequently, cellular damage [27,194]. A recent study by Hull et al. impressively showed that myeloid-specific HO-1 deletion negatively affected renal DC migration to secondary lymphatic organs, and fostered MΦ activation towards a pro-inflammatory phenotype after ischemic injury, which, in turn, impaired renal recovery [199]. In accordance with these observations, the adoptive transfer of HO-1-overexpressing MΦ revealed renoprotective functions during acute renal injury [200].

The role of the iron exporter FPN1, as well as its subcellular location, is still highly debated in the kidney. FPN1 is expressed in MΦ, where it serves as an iron exporter. Its location in tubular epithelial cells is still highly controversial, with some groups suggesting a basolateral expression for iron export to the circulation [194,201], and other groups observing apical expression for iron import of luminal iron [201,202]. Nevertheless, it is clear that MΦ express FPN1 to release iron to their local microenvironment. We and others showed that FPN1 expression is part of the anti-inflammatory MΦ phenotype, adopting iron-releasing functions and, consequently, promoting not only cellular proliferation [196,203] but also T-cell activation [204,205]. With regard to the kidney, more detailed functional investigations using cell-specific knockout models are urgently needed. FPN1 expression may be controlled by the hormone peptide hepcidin, which induces FPN1 degradation and leads to iron sequestration in MΦ. Interestingly, higher serum hepcidin levels were found in acute renal injury, but did not correlate with clinical patient outcome. However, a variety of studies revealed that hepcidin has renoprotective functions [206,207]. These observations allow for the hypothesis of local effects; i.e., by controlling FPN1 levels within the renal microenvironment, modulating FPN1 expression and function, and thereby controlling the MΦ phenotype. The mechanisms by which hepcidin exerts its protective roles in the kidney have not been yet fully elucidated, and urgently need further investigations.

In conclusion, recent advances in elucidating renal iron handling point to a pivotal role of iron in a variety of mechanisms described for renal acute and chronic pathologies. Iron seems to be a critical factor during early phases of renal injury with subsequent recovery, as well as during the transition and progression of chronic disease. Moreover, iron metabolism of different renal compartments needs further attention, especially the role of the MΦ iron phenotype during the progression of the different phases of renal pathologies. A closer look at the iron balance within the kidney may pave the way towards novel therapeutic avenues for treating kidney disease and its complications.

## 6. Lung

### 6.1. MΦ Populations in the Lung

In addition to the structural and functional cells that comprise lung tissue, immune cells are an integral part of the lung and are constantly in flux [208,209,210]. As an organ that has contact with the outside environment, innate immune cells surveil to protect against pathogens and prevent tissue damage. The lungs contain most of the MΦ in the body, and MΦ are the most common immune cell in the lungs. Expectedly, MΦ are generally the first to encounter any kind of external challenge [211,212]. The contact that MΦ maintain with the epithelial layer is vital for reciprocal communication and lung tissue homeostasis. Epithelial-bound MΦ have low phagocytosis activity and cytokine expression in the steady state, yet have the capacity to rapidly initiate inflammatory attacks in response to danger cues from the surrounding microenvironment [213,214]. As MΦ patrol, they also aid in maintaining lung surfactant, and perform functions in identifying, removing, or processing pathogens, harmful particulates, and noxious gases [215,216,217,218,219].

Within the cycle of MΦ recognition, initiation, and participation in an inflammatory attack, MΦ also orchestrate resolution of inflammation within the lungs. Phagocytosis of invading pathogens or debris activate MΦ to secrete oxidative species aimed at invading pathogens, a mechanism that is tightly regulated to limit host-tissue damage. Propagation of inflammatory responses, by release of cytokines, chemokines, or oxidative species, results in the rapid influx of other innate immune cells, like neutrophils, monocytic-derived MΦ (MDMs), eosinophils, and monocytic-derived dendritic cells (MDDCs). Negative feedback loops to reduce inflammatory responses in MΦ initiate the clearance of dead cells or debris, which goes hand in hand with driving the process of tissue remodeling and repair. CD206+ MΦ coordinate this function by secreting TGFβ, IL-13, and IL-4, and the expression of resolution markers MerTK and CD163. The specific timing, intricate cocktail of environmental cues, coordination from other cells, and intracellular signaling pathways involved in MΦ switching from an inflammation-inducing response to a wound-healing and resolution response is still not clear [220,221,222,223,224,225].

All types of MΦ within the lungs share general functional capacities and are found to express classical “MΦ” identifiers, such as CD64+, F4/80+, and CD36+. They also have the capacity to phagocytose, express Fc receptors, and flexibly respond to microenvironmental stimuli [226]. However the population of lung MΦ are heterogeneous in origin and phenotype [227,228]. There are two main types of lung MΦ, alveolar and interstitial. Alveolar MΦ (AMs) occupy the structural components of the alveoli, are densely populated in the lung, and are easily isolated; they are identified using cell-surface markers such as CD45+/SiglecF+/CD11c+, and by oxidative phosphorylation metabolism signatures. They can be further classified into two groups: resident AMs (rAMs), which are derived from embryonic development stage of the body; or the monocytic (or recruited) AMs (mAMs), which possess slight differences from rAMs in terms of cell proliferation and metabolism [228]. Both types of AMs are involved in the maintenance of lung surfactant and engage in defensive roles due to their location within the alveoli.

Non-alveolar MΦ within the interstitum are labelled as interstitial MΦ (IM) [226,229]. Since their abundance is relatively low (~8 times less than AMs), isolation and identification of IMs from the lungs require tissue digestion followed by phenotyping with a diverse combination of cell-surface markers that differ from those found on AMs [230,231]. In-depth transcriptional analyses have identified five subsets of IMs under normal circumstances: IM1, IM2, IM3, IM Lyv1^lo^MHCII^hi^, and IM Lyv1^hi^MHCII^low^. Overall, the function of IMs is generally thought of as regulatory, but in-depth characterization is required for differentiating functional differences between the IM subsets [226,232]. Each category of IMs is determined by the degree of marker expression found on the cell surface, as well as by the specific location where they are found within the lungs. For example, the Lyv1^lo^MHCII^hi^ subset possess a strong antigen presentation cell (APC) function, and can be found at or near nerves within the bronchi. Lyv1^hi^MHCII^low^ secrete cytokines that facilitate repair and can be found around vessels. IMs were originally thought to stem from a putative pool of circulating systemic monocytes, but experiments depleting blood monocytes by the injection of clodronate-containing liposomes showed little to no impact on the population of IMs in the lung [230]. Using comprehensive transcriptomic techniques, other reports suggest that the IM3 subtype is monocytic-derived and therefore recruited, whereas the other IM subtypes are residential. The identification of IM subsets’ cell of origin, as well as functional capacity, is currently being investigated and will likely require advanced techniques of flow cytometry combined with single-cell sequencing [101,228,231,233,234].

### 6.2. Iron and Lung MΦ

As the first responders within the alveolar space, MΦ react rapidly to alterations in iron levels. Accumulation of iron in MΦ occurs mainly to prevent tissue from experiencing iron-induced oxidative stress, and secondly to prevent invasion of pathogens [235], illuminating their important protective mechanism. Under conditions of iron overload, iron can accumulate in structural cells such as alveolar type II cells, vascular smooth muscle cells, and ciliated airway epithelial cells, but the extent to which this occurs is significantly less compared to MΦ. AMs constitutively express TFR1 to promote uptake of Tf- bound iron, but they also import other sources of iron through receptor-specific mechanisms, including lactoferrin (LfR) and DMT1, or through scavenging/phagocytosis of non-Tf-bound sources of iron [236,237,238,239]. AMs are often found with little to no FPN1 and high ferritin light chain expression, indicating a general iron-sequestration phenotype [238]. Indeed, global analysis of lung AMs showed that a proportion are iron-loaded under normal conditions.

The most abundant source of iron for lung tissue originates from the serum [240]. Other sources of iron can be introduced externally, such as by heavily polluted air, and pose significant threats to the integrity of the lung tissue. Chronic exposure usually coincides with adverse side effects including oxidative stress or inflammation, which can culminate in fibrosis or other more major comorbidities. Mining dust, cigarette smoke, and pollution contain aerosolized heavy-metal particulates that collect in MΦ, which can impair MΦ function or result in MΦ apoptosis. Additionally, polluted air can also contain high amounts of noxious and gaseous pollutants, which has been found to suppress phagocytosis in MΦ.

Iron dysregulation in lung tissue has been implicated in many lung diseases. However, the degree to which iron dysregulation is either a driver or a repercussion of lung disease is under intense investigation. Abnormally high levels of iron-loaded MΦ Phhave been observed in patients with idiopathic pulmonary fibrosis (IPF), asthma, chronic obstructive pulmonary disease (COPD), and cystic fibrosis, as well as in patients who smoke [239,241,242]. Furthermore, in patients with asthma and COPD, the number of iron-loaded MΦ has been found to correlate with disease severity [239]. This suggests that there exists a threshold of iron regulation, and thus protection, overseen by AMs, which when surpassed can inundate lung tissue homeostasis and lead to severe lung disease. Indeed, when lung tissue was overwhelmed by iron accumulation alone, key features of asthma were recapitulated in mice, suggesting that dysregulation of iron could be a pathological component for this disease [236,243]. The cause of iron dysregulation in these patients was suggested to be due to a subset of AMs that are dysfunctional/non-functional in iron handling (increased numbers of TFR1+ AMs), and were identified as a significant factor that aggravates IPF in patients [236]. More recent work has provided mechanistic detail by showing this subtype has a skewed phenotype that has both pro-inflammatory and anti-inflammatory features, which function to both produce inflammatory cytokines and facilitate fibrosis. This effect was ameliorated by removing iron with iron chelation in house dust mite (HDM)-induced models of experimental asthma, which indicates a potential therapeutic avenue for future development [243]. The cause of the dysfunction in AMs and the origin of this subtype require further investigation.

Another lung disease that harbors complex MΦ phenotypes that engage in iron dysregulation is non-small-cell lung cancer (NSCLC). Many studies have identified tumor-associated MΦ (TAMs) as anti-inflammatory, and large proportions of this phenotype correlate with a worse prognosis in patients [239]. Anti-inflammatory MΦ have been found to possess high levels of FPN1, which correlates with observations that anti-inflammatory TAMs aid in the growth and development of tumors in NSCLC. MΦ of monocytic origin are more commonly identified as TAMs in lung cancer [244], whereas AMs have only recently been implicated [236]. More recent work has observed that iron accumulation within TAMs of the tumor microenvironment correlates with positive patient outcome compared to those without [245]. In experimental studies and in other cancer types, iron-loaded MΦ have been found to engage in tumoricidal and inflammatory actions, leading to reduced cancer cell growth and number [246]. To target TAMs within the TME of NSCLC provides an interesting avenue of research and drug development for iron-based therapeutics that target TAMs within the TME.

## 7. Brain and Nerves

### 7.1. The MΦ–Iron Liason in the Central Nervous System (CNS)

The brain consumes the body’s energy, and accordingly has a high demand for iron. Iron exerts a well-established role in several physiologic processes such as ATP production, oxidative metabolism, myelination, and synthesis of neurotransmitter, making it an essential protein cofactor for brain functions [247]. Alteration of brain iron homeostasis correlates with different pathologies [248]. Iron deficiency has been associated with cognitive deficits, whereas iron excess leads to neurodegenerative diseases as well as neuroinflammation [249]. In healthy conditions, the blood–brain barrier (BBB) strictly regulates the brain’s iron uptake, protecting the brain from fluctuations in blood iron levels [250]. In addition, it prevents the infiltration of peripheral MΦ into the brain. Traumatic brain injury, inflammation, or late-stage disease states can lead to a disruption of the BBB, thereby allowing monocyte-derived MΦ to infiltrate, as well as the accumulation of iron, finally leading to ROS production and cellular damage [251,252].

Microglia are tissue-resident MΦ that represent the primary innate immune effector cells of the CNS. They are primarily involved in immune/neuroinflammatory responses and regulation of brain homeostasis, but they also exert several other functions, such as neurogenesis, synaptic pruning and plasticity, myelin repair, and oligodendrocyte maturation [253]. Many of these functions require iron as a co-factor. Furthermore, microglia have been shown to regulate brain iron homeostasis by uptaking and storing iron within ferritin [254]. In healthy brain, microglial cells are spread throughout the entire brain parenchyma and are highly ramified, and their processes constantly scan the environment to check for brain damage. In the context of neural injury, microglia became activated, showing morphological and immunophenotypic changes. In response to alterations of the surrounding microenvironment, microglia respond dynamically by polarizing across a spectrum of pro- to anti-inflammatory states [255]. Activated microglia proliferate, and change function, morphology, motility, and glycolytic metabolism [256,257]. Microglia are activated in response to different stimuli and acquire an pro- or anti-inflammatory phenotype. In the pro-inflammatory state, microglia secrete pro-inflammatory cytokines and chemokines such as TNFα, IL-1β, and CCL2, and express iNOS, leading to accumulation of nitric oxide and neurotoxicity. Anti-inflammatory microglia tend to resolve inflammation by releasing IL10, TGF-β, BDNF, and other anti-inflammatory cytokines and trophic factors [258].

Interestingly, inflammatory cytokines have been shown to alter iron uptake and metabolism in microglia cells. Indeed, pro-inflammatory microglia increase the expression of DMT1 and are able to uptake NTBI. They show increased ferritin and labile iron pools. On the other hand, anti-inflammatory microglia increase the expression of transferrin receptor to support increased transferrin-bound iron uptake by receptor-mediated endocytosis. This mechanism may support the increased heme production by mitochondria [247]. Microglia have an important role in maintaining brain iron homeostasis. During development, microglia store iron when myelination is not active, and transfer iron to oligodendrocytes when the myelination proceeds [259]. Iron accumulation in microglia has also been observed in different neurodegenerative diseases, among others Alzheimer’s disease (AD), Parkinson’s disease (PD), and multiple sclerosis (MS) [260,261,262].

All these neurodegenerative diseases are characteristic of the elderly, and it was demonstrated that the brain iron levels increase with age. In particular, in AD, iron accumulation was seen to correlate with amyloid plaques and with neurofibrillary tangles inside the neurons, the two main hallmarks of AD [263]. Interestingly, the brain iron content also was increased in a mouse model of AD as compared with wild-type mice [264]. A recent study [265] correlated brain iron levels in Alzheimer’s patients with a faster decline of memory and cognitive functions.

In neurons, iron homeostasis is strictly regulated at the transcriptional level of mRNA and proteins related to iron metabolism such as Tf, FPN1, TfR1, and ferritin [266]. Furthermore, hepcidin binds FPN1 and mediates its degradation, reducing export of iron from neurons and astrocytes. In addition, ß-amyloid precursor protein (APP) and Tau are also involved in iron regulation, mainly interacting with FPN1. Indeed, APP or Tau knockout mice showed age-dependent brain iron accumulation, indicating the participation of these proteins in iron homeostasis [267].

One of the hallmarks of Parkinson’s disease (PD) is the presence of Lewy bodies in different brain areas, in particular the substantia nigra (SN). Several studies using post-mortem tissues, as well as non-invasive examination of patients, showed increased iron content in SN of PD patients compared with healthy controls [268,269,270]. Furthermore, iron-binding proteins such as ferritin and neuromelanin are decreased in the SN of PD patients [271]. Increased expression of the iron import transporter DMT1 and IRP, as well as decreased expression of FPN1, lead to iron dyshomeostasis in PD patients [272,273]; while α-synuclein aggregation, oxidative stress, and mitochondrial dysfunction, together with iron accumulation, originate a positive feedback loop leading to neuroinflammation and neurodegeneration [274,275].

Neurodegenerative diseases are also characterized by neuroinflammation. Reactive microglia are the main driver of brain inflammation due to a massive production of pro-inflammatory cytokines, ROS, and reactive nitrogen species (RNS), leading to disruption of iron metabolism, mitochondrial dysfunction, and finally to neurodegeneration [274]. Activation of microglia induces iron accumulation by upregulating DMT-1 via pro-inflammatory cytokines of the NFкB mediated transcriptional pathway, and downregulating cell surface expression of FPN1 via hepcidin-mediated internalization, thereby decreasing iron efflux from cells [276]. An increased iron content alters the physiological responses of microglia, leading to increased release of pro-inflammatory cytokines such as TNF-α and interleukin IL-1β [254], as well as promoting free radical formation [277].

Multiple sclerosis (MS) is a chronic neuroinflammatory disease characterized by demyelization and axonal damage with progressive loss of white and gray matter. Even if it is well established that microglia play different roles in MS, spanning from being the driver of inflammation to having important roles in remyelination and in limiting inflammatory responses, it is not clear if their behavior depends on iron homeostasis [278]. Previous studies have functionally linked oxidative damage to axonal and neuronal pathology in MS [279], as well as the involvement of immune cells, in particular activated microglia, in myelin and axonal damage [280]. A recent study shows that microglia isolated from a mouse model of MS or from patients have different phenotypes according to their association with MS lesions. In particular, it was shown that activated microglia associated with active lesions in one of the most severe forms of MS (secondary progressive MS) show changes in iron metabolism, with consequent iron accumulation inside the cells. These alterations lead to oxidative stress and finally to an altered inflammatory phenotype in microglial cells.

Cellular death contributes to alterations of brain function in neurodegenerative diseases. Ferroptosis is a death mode recently described that depends on iron. Ferroptosis is characterized by iron-dependent lipid peroxidation and ROS production. Signs of ferroptosis were seen in many neurodegenerative diseases, including MS, AD, and PD. Mice lacking GPX4, an antioxidant enzyme, show features of ferroptosis and hallmarks of AD, and this phenotype can be reverted using iron chelators. Based on these observations, iron chelation can be used as therapeutic approach to ameliorate Alzheimer’s symptoms. A phase II clinical trial of the use of iron chelators in Alzheimer’s patients began in early 2021. Interestingly, genetic or pharmacological iron chelation also appears to be promising for PD, both in a mouse model of PD and in clinical trials [281,282].

Iron brain accumulation during aging has been associated not only with neurodegenerative disease, but also with an increased severity and poor prognosis for brain tumors. Cancer cells frequently show alterations of expression of proteins involved in iron homeostasis, such as upregulation of TfR1, Tf, or ferritin; and downregulation of FPN1. Glioma is the most common brain tumor both in adults and children. Up to 50% of the cellular content is composed of infiltrating non-cancerous cells, mainly microglia and circulating MΦ [283]. These glioma-associated microglia and MΦ (GAM) are recruited by glioma cells, secreting several chemoattractants such as CCL2, Cx3CL1, SDF-1, and CSF-1, among others [284]. Once recruited to the glioma site, GAM acquire a pro-tumorigenic phenotype, secreting anti-inflammatory cytokines such as TGFβ and IL-10, as well as angiogenic factors such as VEGFα [284]. Furthermore, the tumor milieu promotes epigenetic and transcriptional programs that create new molecular identities of the GAM critical for glioma progression [283]. In recent years, the avenue of single-cell transcriptomic analysis has revealed a strong heterogeneity of GAM, whereby their function in the development and progression of glioma is still not fully understood [285]. A better knowledge of this process and transcriptional signature will help to find new targets for glioma therapies. Recently, Shonberg et al. (2015) characterized a specific population of cancer cells inside the glioma, the cancer stem-like cells (CSC). These cells have properties of stem cells, they survive in unfavorable conditions such as a lack of nutrients or hypoxia, and are chemotherapy resistant. They correlate with a poor prognosis. Their proliferation is supported by a mechanism of iron scavenging: they express high levels of Tf and TfR1, as well as ferritin. Knocking down ferritin reduced the upregulation of TfR1 and the proliferation of CSC. Furthermore, reducing ferritin expression increased chemotherapy sensitivity of CSC. Interestingly targeting ferritin also is beneficial in other types of cancer. Specifically targeting iron availability to CSC appears to be a more selective therapy against glioma [286].

To overcome the two main barriers to delivery of therapeutics into the brain to treat brain disorders, namely the sensitivity of nerve cells and the BBB, different strategies have been developed in recent years. Among others, Tf and TfR have been used as drug-delivery systems across the BBB. Several types of cancer cells express high levels of TfR, so drugs directly bound to Tf or on antibodies against TfR are mainly targeting malignant cells, resulting in a reduction of tumor growth. Binding drugs to Tf or antibodies against TfR also has been used as a strategy to cross the BBB due to the high expression of TfR on endothelial brain cells. However, Tf is not a good drug carrier due to its rapid turnover, and strategies to increase its stability and drug delivery are under study [287]. After spinal cord injury (SCI), microglia cells, as well as circulating MΦ, are recruited to the lesion site. At the beginning, they acquire a pro-inflammatory phenotype, with massive phagocytic potential to clean the injured site of debris and dead cells, then the anti-inflammatory microglia and infiltrating MΦ promote repair and regeneration [288,289]. Myelin phagocytosis promotes a MΦ switch from pro-inflammatory to anti-inflammatory polarization in order to sustain recovery. Most MΦ after SCI maintain a pro-inflammatory polarization that interferes with the recovery process. Kroner et al. showed that this phenomenon is due to the high intracellular content of iron as a consequence of red blood cell phagocytosis. High iron induces the expression of TNFα, which is known to block the transition from pro-inflammatory to anti- inflammatory polarization [290]. Dysregulation of iron metabolism and altered expression of iron regulatory genes were shown after SCI, and iron chelation was proposed as therapy to improve recovery after SCI.

Spinal cord microglia also are activated after peripheral nerve lesions due to the release of CSF-1 at the dorsal horn level from the central terminal of damaged primary sensory neurons. This activation is important for the development and maintenance of neuropathic pain [291]. Interestingly, it was shown that in a mouse model of sickle cell disease, the elevated free heme content in spinal cord tissue due to chronic hemolysis mediates evoked pain hypersensitivity via TLR-mediated activation of microglia [292].

### 7.2. MΦ Populations in the Peripheral Nervous System (Ganglia and Nerves)

Much less is known about peripheral nervous system-associated MΦ. Ganglia and nerves are not isolated by the BBB, so it is difficult to distinguish between PNS-associated MΦ and circulating ones. A distinct population of PNS-resident MΦ was identified almost 30 years ago [293], but it only recently could be characterized [294,295]. Circulating MΦ are usually not associated with nerves or found into the ganglia; they are recruited and infiltrate the tissue after infection or injury [296]. After peripheral nerve injury, MΦ accumulate at the site of injury within 3 days [296], and are recruited by specific signals like MCP-1, IL-1β, and CCL2. Here, they proliferate and acquire a classically activated phenotype, secrete pro-inflammatory factors, and promote debris removal and elimination of products of Wallerian degeneration of the distal segment of the nerve, as well as apoptotic cells [297]. Once they have cleaned the injury site, MΦ transit from the pro- to the anti-inflammatory state. Alternatively activated MΦ secrete anti-inflammatory cytokines and promote tissue repair [298]. Interestingly, 3 days after sciatic nerve ligation, MΦ infiltrated into dorsal root ganglia (DRG) were mainly of the anti-inflammatory phenotype [299,300,301]. The beneficial or detrimental effect of iron overload in peripheral neuropathy is still debated [302]. Some papers report iron promoting infiltration of anti-inflammatory MΦ and resolution of inflammation [303], while others show a worsening of neuropathy in the presence of high iron content [304,305].

PNS-associated MΦ are present under homeostatic conditions, both in peripheral nerves and in ganglia, and they are self-maintained [306]. Based on global transcriptomic signature, they are MΦ with some properties of CNS microglia, based on both endoneural localization and surface marker expression [294]. Their function in homeostasis and injury is still not fully clarified, but it seems that they contribute to nerve surveillance, sliding along sensory neurons axons [306]. After injury, they have been involved in axon sprouting of sensory neurons [306] and in Wallerian degeneration of the sciatic nerve [296]. Iron-dependent modulations of PNS-associated MΦ functions and/or phenotype are still not identified.

## 8. Conclusions

**Figure 1:**
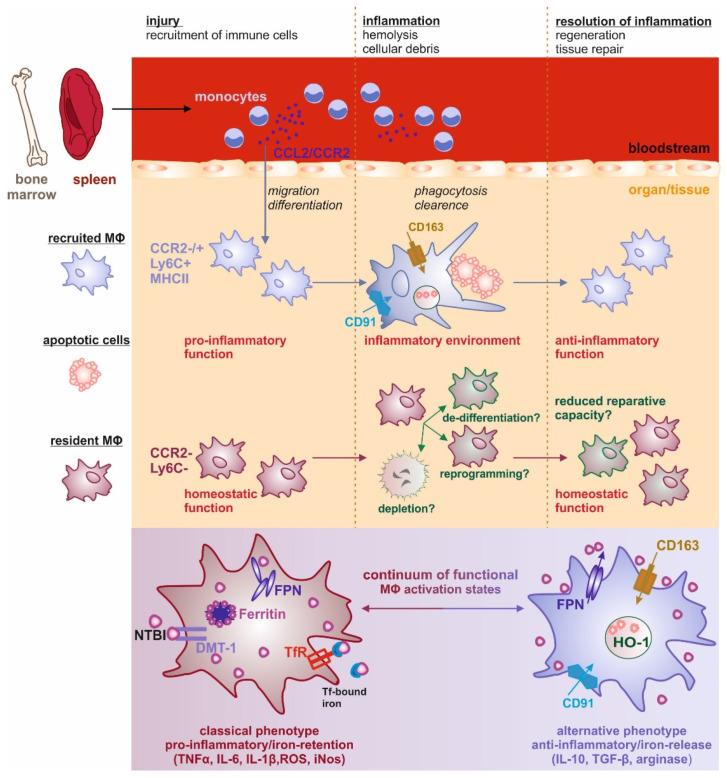
Hypothetical scheme of MΦ iron polarization during injury and recovery. Different MΦ subpopulations have been described with varying levels of lymphocyte antigen 6 (Ly6C) and major histocompatibility complex (MHCII). Under physiological conditions, resident MΦ predominate together with embryonic progenitors that are not replenished by circulating monocytes under steady-state conditions. Resident MΦ in the healthy state promote tissue homeostasis. CCR2^+^ MΦ are replenished by blood monocyte recruitment and local proliferation, whereas CCR2^−^ MΦ are repopulated by local proliferation. Infiltrating pro-inflammatory monocyte-derived MΦ (Ly6C^+^ CCR2^−^ and Ly6C^+^ CCR2^+^) promote tissue injury and death by substitution of the resident MΦ subpopulation. Shown is a continuum of functional activation states, with two extreme phenotypes linked to the highly diverse MΦ functional activation states in the different stages of inflammation and resolution of inflammation. The iron phenotype is also closely related to the MΦ polarization profile, with its two extremes of iron retention and iron release.

The innate immune system plays a crucial role in acute inflammation and resolution of inflammation, but iron handling by MΦ is an often-disregarded feature contributing to physiologic function, and may also be involved in disease progression. With this review, we aimed to contribute to the understanding of iron handling facilitated by MΦ in the systemic and cellular microenvironment, playing a central role in physiologic and pathophysiologic functions. The main points of our review are summarized in Figure 1.

## Data Availability

Not applicable.
